# Two Morphological Awareness Components Have Different Roles in Chinese Word Reading Development for Primary Schoolers

**DOI:** 10.3389/fpsyg.2022.894894

**Published:** 2022-07-04

**Authors:** Hailun Liu, Jing Wang, Lei Wang, Wenjun Zhang, Ciping Deng

**Affiliations:** ^1^Shanghai Key Laboratory of Brain Functional Genomics, Affiliated Mental Health Centre (ECNU), School of Psychology and Cognitive Science, East China Normal University, Shanghai, China; ^2^Shanghai Changning Mental Health Center, Shanghai, China; ^3^Jiangsu Provincial Key Constructive Laboratory for Big Data of Psychology and Cognitive Science, Yancheng Teachers University, Yancheng, China; ^4^Key Laboratory for Biomedical Engineering of Ministry of Education, School of Biomedical Engineering and Instrument Science, Zhejiang University, Hangzhou, China; ^5^Department of Special Education and Counselling, The Education University of Hong Kong, Tai Po, Hong Kong SAR, China

**Keywords:** morphological awareness, general morphological knowledge, morphological meaning analysis, Chinese, word reading

## Abstract

Morphological awareness is multi-factorial by nature and consists of general morphological knowledge and morphological meaning analysis; the first refers to the recognition and manipulation of morphological structures, and the second refers to the inference of word semantics by utilizing morphological knowledge. Contrasting the roles of two morphological awareness components in word reading could help resolve the controversy about whether morphological awareness could independently contribute to Chinese word reading. Thus, the study explored how morphological awareness components contributed to word reading development in Chinese context. A group of 299 Chinese children in grades 3 and 4 were recruited and tested twice with the interval of half a year, by a series of tasks on morphological awareness components, word reading, and some control variables. Results showed that, after controlling for vocabulary and other linguistic variables, morphological meaning analysis could independently predict word reading, whereas general morphological knowledge could only indirectly predict word reading, a process mediated by morphological meaning analysis. This study showed independent contribution of morphological awareness to Chinese word reading development. By clarifying the ways of how different morphological awareness components support children’s word reading development, the findings enhance our understanding about the potential mechanism underlying the relation between morphological awareness and Chinese word reading.

## Introduction

Morphological awareness (MA) is defined as the awareness of morphological structures and the ability to recognize and manipulate morphemes (Kuo and Anderson, 2006). Researchers proposed that MA could be classified into two components: general morphological knowledge and morphological meaning analysis. The former refers to the sensitivity of identifying and manipulating morphological structures, whereas the latter refers to understanding the relation between multi-morpheme words and constituent morphemes (e.g., Carlisle, 2000; Goodwin et al., 2017). Most studies have only measured Chinese MA with general morphological knowledge and have yielded inconsistent results on whether MA could contribute to Chinese word reading independently of vocabulary (e.g., Yeung et al., 2013; Tong et al., 2017). Inspired by several recent findings (Deacon et al., 2017; Levesque et al., 2017, 2019), this study asserted that including morphological meaning analysis in MA measures was reasonable to gain a comprehensive understanding of the contribution of MA to reading acquisition. The present study primarily aimed to distinguish these two MA components and explored which of them (or both) independently contributed to Chinese word reading development after controlling vocabulary.

In addition, studies showed that general morphological knowledge could advance the development of morphological meaning analysis (Zhang et al., 2016; Levesque et al., 2019). Morphological meaning analysis could also possibly contribute to word reading inferred from prior research (e.g., Zhang, 2015; Deacon et al., 2017; Levesque et al., 2019). Furthermore, general morphological knowledge may indirectly contribute to word reading through the mediation of morphological meaning analysis. Thus, the second goal of the present study was to examine the role of morphological meaning analysis in the association between general morphological knowledge and word reading development.

### Relation Between Morphological Awareness and Word Reading

Across languages, studies have found the important role of MA in word reading (e.g., Casalis and Louis-Alexandre, 2000; Kuo and Anderson, 2006; Yeung et al., 2013). In learning to read English, researchers suggest two pathways indicating how MA contributes to word reading, namely, the orthographic–phonological pathway and the orthographic–semantic pathway. *Via* the orthographic–phonological pathway, MA could assist children to parse multi-morpheme words into morpheme units and thus decoding these words efficiently through larger morpheme units, instead of through smaller phoneme units. Through the orthographic–semantic pathway, MA could help children infer the semantics of multi-morpheme words by combining the meanings of constituent morphemes (e.g., Nagy et al., 2013; Kearns and Al Ghanem, 2019). The strengthened semantic representation could promote word reading performance as evidenced by some studies (e.g., Perfetti et al., 2005; Wang et al., 2013), although word reading tasks merely require readers to pronounce words according to orthography. As proposed by the lexical quality hypothesis (e.g., Perfetti, 2007), the representation quality of orthography, phonology, and semantics all could determine word reading performance.

In Chinese reading context, characters are the basic units of written language, and a character simultaneously represents a morpheme and a syllable. For words, 75–80% of them are compounded with two or more characters with the same square of space between each character (Packard, 2000; McBride, 2016). Given the clear boundary between characters, children do not need MA to parse multi-morpheme words, and MA could not facilitate Chinese word reading through the orthographic–phonological pathway (Kuo and Anderson, 2006).

Moreover, Chinese words are relatively semantically transparent, and Chinese characters are highly productive, which enable MA to facilitate word reading through the orthographic–semantic pathway (e.g., Li and McBride-Chang, 2014; Tong et al., 2017). Specifically, with the help of MA (e.g., subordinate structure awareness), children could easily infer the meanings of semantically transparent words, such as “木桌 (/mu4 zhuo1/, wooden table),” if they know the definition of single morphemes such as “木 (/mu4/, wood)” and “桌 (/zhuo1/, table).” Furthermore, once children know the meaning of “桌,” they could utilize MA skills to obtain efficiently the meanings of words, such as “铁桌 (/tie3 zhuo1/, iron table),” “石桌 (/shi2 zhuo1/, stone table),” and “圆桌 (/yuan2 zhuo1/, round table),” which contain the known morphemes. As Li and McBride-Chang (2014) proposed, MA and knowledge of characters are the two prerequisites for Chinese word learning.

### Which MA Components Could Independently Predict Chinese Word Reading?

Although researchers have suggested that MA could contribute to word reading through the orthographic–semantic pathway, the mechanism underlying the MA–Chinese word reading relation has not been well understood (e.g., Chen et al., 2008; Liu et al., 2017). One of the controversial questions regarding the relation between MA and Chinese word reading was whether MA could contribute to Chinese word reading independently of vocabulary. Tong et al. (2017) showed that the contribution of MA to word reading was totally mediated by vocabulary, and MA could no longer predict word reading after controlling vocabulary. Similarly, Hulme et al. (2019) found that MA could not independently contribute to word reading development beyond the controls of vocabulary and other linguistic variables. However, some studies suggested a unique contribution of MA to word reading after controlling vocabulary (e.g., Liu et al., 2013; Yeung et al., 2013). Thus, whether MA’s contribution to word reading depended on vocabulary was unresolved.

Several recent findings on the relation between MA and reading comprehension shed light on whether and how MA contributed to word reading (Deacon et al., 2017; Levesque et al., 2017, 2019). These studies divided MA into general morphological knowledge and morphological meaning analysis and took independent measures of them. Before the review of these studies, we first introduced the two MA components. General morphological knowledge represents children’s awareness of the morphological structures of multi-morpheme words, whereas morphological meaning analysis is an advanced skill of inferring words’ semantic meanings by utilizing morphological structures (Goodwin et al., 2017). The latter MA component has been represented by different terms, such as morphological problem solving (Anglin, 1993) and lexical inference (Zhang et al., 2016). These names all represent the meaning inference process, including the segmentation of words into constituent units, the analysis of the semantic relation between constituent morphemes and whole words, the synthesis of whole word semantics, and the semantic retrieval of single morphemes (Ku and Anderson, 2003; Zhang et al., 2016; Goodwin et al., 2017). Carlisle (2000) first delineated the two components of MA. Moreover, the distinction of the two components has been supported by conducting confirmatory factor analyses for children in grades 3 (Levesque et al., 2017) and 7, 8 (Goodwin et al., 2017).

By using the path model analysis, Levesque et al. (2017) found that after including the measurement of morphological meaning analysis, the mediating role of vocabulary was supplanted by morphological meaning analysis in the relation between general morphological knowledge and reading comprehension. This result indicated that morphological meaning analysis, rather than general morphological knowledge, could contribute to reading comprehension independently of vocabulary. Similarly, Deacon et al. (2017) and Levesque et al. (2019) directly showed that only morphological meaning analysis, but not general morphological knowledge, could uniquely contribute to reading comprehension after controlling vocabulary.

According to the above researchers, morphological meaning analysis represented the online process of inferring word semantics, whereas vocabulary represented the stored knowledge of word semantics. When children have to figure out the meanings of specific words through morphological meaning analysis, they may not have the stored semantic meanings of these encountered words in their vocabulary. Conversely, when children know the meanings of some words in their vocabulary, they may not be able to analyze the semantic relations between whole words and constitute morphemes by using morphological meaning analysis (Anglin, 1993).Therefore, morphological meaning analysis and vocabulary are different constructs, though functionally related. Morphological meaning analysis could then facilitate the semantic understanding of multi-morpheme words independently of vocabulary, which further promoted comprehension. In addition, general morphological knowledge could not necessarily arrive at word semantics, and only morphological meaning analysis has access to word semantics by analyzing morphological structures. The distinct functions of the two components could lead to the result that the latter component, rather than the former one, independently predicted reading comprehension.

Although there are differences between alphabetic languages and Chinese as mentioned, MA could contribute similarly to both English reading and Chinese reading *via* the orthographic-semantic pathway. Therefore, morphological meaning analysis may also play a unique role in Chinese reading, just as it did in English reading. In other words, in Chinese, morphological meaning analysis with stronger word semantic inference ability was also more likely to contribute to word reading independently of vocabulary compared with general morphological knowledge.

However, a number of previous studies on the relation between MA and Chinese reading have measured MA only by compound awareness tasks, which assessed the identification and manipulation of compound structures but not the skill of obtaining word semantics precisely (e.g., Yeung et al., 2013; Tong et al., 2017). Therefore, this kind of tasks evaluated general morphological knowledge but not morphological meaning analysis. The adoption of general morphological knowledge measures may thus lead to the inconsistent findings about whether MA could contribute to word reading independently of vocabulary (Liu et al., 2013; Yeung et al., 2013; Tong et al., 2017; Hulme et al., 2019). To help resolve the dispute concerning the role of MA in word reading, the present study, which focused on both general morphological knowledge and morphological meaning analysis, aimed to examine which of the two components (or both) could contribute to Chinese word reading development independently of vocabulary.

### Mediating Effect of Morphological Meaning Analysis on the Relation Between General MA Knowledge and Word Reading

Regarding the relation between the two MA components, it has been suggested that general morphological knowledge encapsulates foundational skills, which facilitate the development of morphological meaning analysis (Kuo and Anderson, 2006; Goodwin et al., 2017). The suggestion was supported by empirical evidence that the former MA subcomponent could predict gains in the latter one (Zhang et al., 2016; Levesque et al., 2019). When children grasp a good awareness of morphological structures, they also gain an increased likelihood to analyze the relation between words and constituent morphemes *via* using the structure knowledge, which, in turn, improves morphological meaning analysis. Moreover, morphological meaning analysis showed a mediating effect on the correlations between general morphological knowledge and two reading skills (i.e., vocabulary and comprehension; Zhang, 2015; Levesque et al., 2017, 2019). Researchers suggested that general morphological knowledge could boost the development of morphological meaning analysis, which could precisely obtain multi-morpheme words’ semantics and thereby improved vocabulary and comprehension. Apart from vocabulary and reading comprehension, word reading performance could also be improved when words’ semantics are strengthened (Perfetti et al., 2005; Perfetti, 2007). Therefore, morphological meaning analysis may also mediate the relation between general morphological knowledge and word reading.

Few studies have tapped into morphological meaning analysis in Chinese, and we have not yet found any study exploring if morphological meaning analysis mediates the relation between general morphological knowledge and Chinese word reading. This study thus fills the research gap by exploring the issue in Chinese reading context.

### The Measurement of Morphological Awareness Components in Chinese

Homophone/homograph awareness tasks, which are common Chinese MA measures (e.g., Hao et al., 2013; Liu et al., 2017), are likely to capture morphological meaning analysis. In such tasks, children are orally presented with two or more words, such as “春节 (/chun1 jie2/, spring festival)” and “节约 (/jie2 yue1/, save).” Both words contain the same homophones/homographs (such as “/jie2/”), and children need to judge whether the homophones/homographs in different words have the same meaning. Considering that certain meanings of homophones/homographs with multiple interpretations could only be decided when the morphemes appear in words, children may require context information to obtain single-morpheme meanings (Li et al., 2017b). MA involves word formation rules (Kuo and Anderson, 2006) so that it could provide word context information and thereby help retrieve the meanings of homophones/homographs.

Empirical studies also supported that children could retrieve the meanings of constituent morphemes by analyzing words’ morphological structures. Tsang and Chen (2013) found that children could ascertain the meanings of constituent morphemes presented within words by using context information. This finding suggested that MA, as a context clue, was likely to help children determine the meanings of constituent morphemes from words. Moreover, Wang and Liu (2020) found that MA contributed more to character reading for small-family-size morphemes (i.e., characters) than for large-family-size morphemes, and suggested that MA could facilitate character reading by retrieving word context information to indicate single-morpheme meanings. In this study, we named the ability to retrieve homophones/homographs’ meanings from words’ meanings as morphological retrieval skill. The skill includes the segmentation of constituent morphemes and the retrieval of constituent morphemes’ meanings by analyzing morphological structures (e.g., Hao et al., 2013). Thus, the skill belongs to morphological meaning analysis, according to the definition by Goodwin et al. (2017).

However, we argue that homophone/homograph awareness tasks in previous studies did not always capture the meaning-analysis morphological retrieval ability for three reasons. First, homophone/homograph awareness tasks tend to capture the morphological retrieval ability only with high-frequency word items. In mental lexicon, high- and low-frequency words tend to be represented in holistic and decomposed forms, respectively (e.g., Zhang and Peng, 1992). For low-frequency items, children have decomposed word representations and could directly understand the constituent-morpheme meanings, which possibly makes performances on the homophone/homograph awareness tasks reflect the knowledge of single morphemes. Only when the items are high-frequency will children have the whole word representations, in which situation they could use the morphological retrieval ability to obtain the constituent morpheme meanings from whole words. Therefore, homophone/homograph awareness tasks tend to emphasize awareness of specific morphemes rather than MA knowledge, as suggested by researchers (Liu and McBride-Chang, 2010; Li and McBride-Chang, 2014). As far as we know, only two studies used familiar and high-frequency word items in Chinese homophone/homograph awareness tasks (Hao et al., 2013; Li et al., 2017a), whereas others did not control words’ frequency or familiarity and may not capture the morphological retrieval ability.

Second, homophone/homograph awareness tasks are not suitable for measuring young children’s morphological retrieval ability. As studies showed, young children, such as kindergarteners and early-stage primary schoolers, performed poorly in retrieving single-morpheme meanings from words (Krott and Nicoladis, 2005; Hao et al., 2013; Li et al., 2017a). The reason for this phenomenon probably lies in that young children initially learn the holistic representations of words in oral language and could only learn to segment words when they are older (e.g., Taft, 1985; Packard, 2000).

Third, the acquisition of orthographic representations, which helps children distinguish homophones and thus reduce task difficulty, may confuse measurement results. Li et al. (2017a) found that primary school children could better distinguish homophones in the pair of “高山 (/gao1 shan1/, high mountain)” and “糕点 (/gao1 dian3/, pastries)” than homographs in the pair of “面包 (/mian4 bao1/, bread)” and “面孔 (/mian4 kong3/, face).” This finding suggested that adopting homographs as items would avoid confusing effect of orthographic representations on the assessment of the morphological retrieval ability.

To measure morphological meaning analysis accurately, the study selected only high-frequency words containing homographs as task items. We chose grades 3 and 4 children as participants because grade 3 is the transition from “learning to read” to “reading for meaning” (Chall, 1983); hence, students above grade 3 may be able to analyze the semantics of morphemes. For general morphological knowledge, we used compound awareness tasks as the measurement, because compound structures are the most dominant morphological structures and they are the most productive word formation rules in Chinese (Packard, 2000).

### The Present Study

The current study aimed to adopt both general morphological knowledge and morphological meaning analysis, and explored the contributions of different MA components to Chinese word reading. Additionally, using the longitudinal studies has profound theoretical significance to the precise models of reading development, by providing insight into the mechanism of the MA–reading relation. In particular, it was still under debate whether MA could predict gains in Chinese word reading. Evidence suggested the contribution of early MA to later word reading when controlling for the autoregressive effect of reading skills (Yeung et al., 2013; Lin et al., 2019), whereas other researchers failed to demonstrate the predictive role of MA (Tong et al., 2009; Wang et al., 2015; Hulme et al., 2019). Thus, a short-term longitudinal design was used to examine the developmental relation between MA and word reading.

First, this research aimed to explore which MA components contributed to Chinese word reading development independently of vocabulary. Compared with general morphological knowledge, as reviewed above, morphological meaning analysis has more direct access to semantic representations. We therefore hypothesized that morphological meaning analysis could contribute to word reading development, independently of vocabulary and general morphological knowledge.

If the first hypothesis was supported, we furthermore hypothesized morphological meaning analysis mediated the contribution of general morphological knowledge to word reading development. The second question was to examine the role of morphological meaning analysis in the relation between general morphological knowledge and word reading development.

We used an autoregressive path model to explore the roles of MA components in Chinese word reading development with a sample of children in grades 3 and 4, the stage when they could be likely to possess morphological meaning analysis skills (Chall, 1983). Gender was controlled, because studies consistently found that girls outperformed boys in reading achievement (e.g., Logan and Johnston, 2010). Age was also controlled, given that children rapidly develop literacy skills in their elementary school years (e.g., Hao et al., 2013). In addition, variables of children’s vocabulary, orthographic knowledge, radical awareness, phonological awareness, rapid automatized naming, and nonverbal intelligence, which all have effects on word reading (e.g., Tong et al., 2009; Yeung et al., 2013), were controlled in the model.

## Materials and Methods

### Participants

Participants were 309 third or fourth graders, with none experiencing any intellectual sensory and/or behavioral difficulties (according to their teachers’ report). They were recruited from two public elementary schools in a major city of Mainland China, in which most of the children came from families of middle socioeconomic background. Ten students missed the test at the second time point, final analyses thus included 299 children who participated in both tests, with 155 in grade 3 and 144 in grade 4 (75 and 78 boys in the two grades, respectively). Mean ages are in Table 1.

**Table 1:** Means and standard deviations for all measures among two graders.

Measures	M	SD	Range	Skewness	Kurtosis
T1 Age (in years)	9.22	0.60	8.23–10.38	0.12	−1.32
T1 General morphological knowledge	96.53	10.76	59.00–119.00	−0.52	0.17
T1 Morphological meaning analysis	16.88	2.23	5.00–20.00	−1.39	3.62
T1 Word reading	139.64	16.92	78.00–199.00	−0.29	0.63
T1 Vocabulary	41.21	6.41	20.00–58.00	−0.37	0.46
T1 Orthographic awareness	35.71	2.71	25.00–40.00	−1.20	2.22
T1 Radical awareness	31.12	3.04	18.00–37.00	−0.73	0.86
T1 Phonological awareness	21.33	3.24	5.00–28.00	−1.13	2.40
T1 Rapid naming	2.70	0.51	1.40–4.71	0.70	0.93
T1 Nonverbal intelligence	52.24	9.25	12.00–71.00	−0.88	1.83
T2 General morphological knowledge	103.80	9.67	60.00–123.00	−0.84	1.42
T2 Morphological meaning analysis	17.23	1.91	10.83–20.00	−0.98	0.99
T2 Word reading	145.22	16.82	86.00–188.00	−0.12	−0.01

T1 represents time 1; T2 represents time 2.

### Measures

#### General Morphological Knowledge

General morphological knowledge was based on Liu and McBride-Chang (2010) and had been used in several previous studies to assess compound awareness (e.g., Liu et al., 2017; Wang and Liu, 2020). This task was orally presented with 31 items. Children were asked to orally create novel words by combining acquired morphemes according to the description of an object or concept. For example, 我们把专门用来切石头的刀叫做什么? “What should we call a knife used for cutting stones?” The answer was切石刀 (/qie1shi2 dao1/, stone-cutting knife). Children’s answers were rated on a 0- to 4-point rating scale on the basis of their knowledge of morphemes and morphological structures. A 4-point answer included all the critical morphemes with a correct and succinct structure; 3 points were given to answers including redundant morphemes but applying a correct structure; 2 points were given to answers which missed critical morphemes and resulted in incomplete structure; 1 point was given if responses included some of the critical morphemes but with incorrect structures; 0 point was allocated for unrelated responses or no response. In the pre-test, 5 children in grades 3 who did not participate in the formal test rated words’ familiarity, and the result showed that they were familiar with the presented words. The internal consistency reliability for this test in the present study was adequate (Cronbach’s α = 0.75).

#### Morphological Meaning Analysis

The morphological retrieval task was used to measure morphological meaning analysis. In this task adapted from Shu et al. (2006), children were orally presented with pairs of two-morpheme words. Each pair such as草地 (/cao3 di4/, grassland) and草率 (/cao3 shuai4/, sloppy) contained one homograph (e.g., 草/cao3/, grass), and children were asked to answer whether the homographs in the two words had the same meaning or not. Children could also answer “I do not know” to minimize guessing. A total of 50 pairs of high frequency (> 80 per million) and transparent words were selected from a first-grade Chinese textbook. Words’ semantic transparency was rated by the average contribution of each constitute morpheme’s meaning to the whole word’s meaning (with the total score of 10 for each morpheme; Tsang and Chen, 2013). In the present study, the average score of the contributions for constitute morphemes was near to 5, showing that the selected words were semantically transparent.

In addition, the semantic relatedness of whole words could ease the task (Hao et al., 2013; Li et al., 2017a), but word pairs in the “YES” condition tend to be semantically similar owing to semantic transparency. To ensure that it was challenging enough for the students in the “YES” condition, we prioritized word pairs with different whole word semantics, such as 晚会 (evening party) and 晚报 (evening paper). Finally, 20 pairs of items (9 and 11 pairs in the “YES” and the “NO” condition, respectively) with appropriate difficulty (approximately 3 points on a 0- to 5-point rating scale) for children were selected, according to the difficulty evaluation in the pre-test. The internal consistency reliability for this test in the present study was acceptable (Cronbach’s α = 0.61).

#### Word Reading

Word reading was based from previous studies (Deng et al., 2015; Wei et al., 2015). Children were asked to read aloud 220 Chinese two-character words arranged in terms of increasing difficulty and stopped after 15 consecutive errors. A participant’s score was the total number of correctly read Chinese words. The internal consistency reliability for the test in the present study was excellent (Cronbach’s α = 0.92).

#### Vocabulary

We assessed vocabulary using the vocabulary component of the Chinese version of the Wechsler Intelligence Scale for Children (WISC-IV; Zhang, 2009). Children were required to explain the definitions of the orally presented words (objects or concepts). They obtained 2 points for accurate descriptions or synonyms, 1 point for related answers and 0 point for wrong definitions or no response. Testing stopped if children scored 0 on four consecutive items. In the present study, the internal consistency reliability for this test was adequate (Cronbach’s α = 0.81).

#### Orthographic Awareness

The orthographic awareness was assessed by the pseudo-character judgement subtest (Wang et al., 2011). The task was to distinguish whether the characters were true or false. A total of 20 low-frequency characters (e.g., 謇) and 20 matched pseudo-characters (e.g., 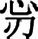) which have the legal components but in illegal positions were used. In the present study, the internal consistency reliability for this test was adequate (Cronbach’s α = 0.70).

#### Radical Awareness

The radical is a graphic component of the character which may have semantic or phonetic indication to the character. This measurement was adapted from the semantic picture-naming task from Tong et al. (2017). A total of 37 target pseudo-characters consisting of legal radicals in legal positions were set (e.g., 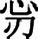). After the target character, each item also consisted of four colorful pictures, and children were required to choose the picture that could best depict the meaning of the target character. For example, the four pictures corresponding to the character “
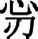
” represented a watermelon, a flower, a dragonfly, and a fish, respectively. The answer in this example was the picture of a fish because the semantic radical of the target character was “鱼 (fish).” The internal consistency reliability for this test in the present study was adequate (Cronbach’s α = 0.74).

#### Phonological Awareness

Phonological awareness was tested using the phoneme deletion task (Xue et al., 2013). Children were orally presented one monosyllable each time and were asked to say aloud the syllable without one phoneme. Given the syllable of/mei4/, after taking away the initial phoneme /m/, the answer would be/ei4/. The internal consistency reliability for this test in the present study was 0.74.

#### Rapid Automatized Naming

Rapid automatized naming (RAN) was assessed with Digit naming which was adapted from Hong Kong Test of Specific Learning Difficulties in Reading and Writing (Ho et al., 2000). The digits 2, 4, 5, 7, and 9 were repeated ten times and arranged in semi-random order in a 5 × 10 matrix. Children were required to name them as fast as possible from left to right and from top to bottom. They named the matrix twice, and the time and errors in naming all stimuli in each time were recorded. The score was calculated by dividing the average number of correctly named items by the average time in the two matrices. The internal consistency reliability for this test in this study was 0.82.

#### Nonverbal Intelligence

We assessed children’s nonverbal intelligence with the Chinese Combined Raven’s Test (CRT) revised by Li et al. (1988). The task composed of 72 items, and children were required to choose the best one to fill the missing part of a matrix from 6 options in approximately 40 min. A participant’s score was the total number of correct answers (max = 72). The reliability for this test from the manual is 0.95.

### Procedure

The first test for children was conducted in the first semester of grades 3 and 4 (in November), and the second was in the next semester (in May). Individual tests entailed the two MA tasks, word reading and the control measures (vocabulary, orthographic awareness, radical awareness, phonological awareness, RAN, and nonverbal intelligence). The majority of tasks were tested individually in a quiet room by trained experimenters, except for nonverbal intelligence, which was administered in groups. Task administration for each child lasted for approximately 1.5 h and was broken up into two or three shorter sessions on the bases of children’s attention span. All procedures in the study were beforehand approved by the University’s Research Ethics Committee. Consent was obtained from all participants and their parents before testing.

## Results

### Preliminary Analysis

Table 1 presents the descriptive information. The result showed that the absolute values of skewness for all measures were within 3 (kurtosis values within 4), indicating that these variables were normally distributed (e.g., Hair et al., 2010). Tables 2, 3 display zero-order correlations among all variables across grades. Specifically, the correlation between general morphological knowledge and morphological meaning analysis was below 0.46 across samples from the two grades, suggesting that the two MA components were not measuring identical skills.

**Table 2:** Correlations between all measures among combined graders at Time 1 and Time 2.

S. No.	Variables	1	2	3	4	5	6	7	8	9	10	11	12	13	14
1.	T1 General morphological knowledge	–													
2.	T2 General morphological knowledge	0.45^**^	–												
3.	T1 Morphological meaning analysis	0.36^**^	0.30^**^	–											
4.	T2 Morphological meaning analysis	0.40^**^	0.39^**^	0.41^**^	–										
5.	T1 Word reading	0.37^**^	0.31^**^	0.34^**^	0.35^**^	–									
6.	T2 word reading	0.39^**^	0.34^**^	0.39^**^	0.41^**^	0.88^**^	–								
7.	Gender	−0.08	0.01	−0.02	−0.04	−0.06	−0.08	–							
8.	T1 age	0.07	0.06	0.09	0.06	0.34^**^	0.31^**^	−0.06	–						
9.	T1 Vocabulary	0.27^**^	0.27^**^	0.32^**^	0.30^**^	0.55^**^	0.54^**^	−0.01	0.34^**^	–					
10.	T1 Orthographic awareness	0.07	0.03	0.18^**^	0.04	0.15^*^	0.12^*^	0.02	0.02	0.10	–				
11.	T1 Radical awareness	0.19^**^	0.20^**^	0.19^**^	0.16^**^	0.34^**^	0.30^**^	−0.02	−0.04	0.12^*^	0.13^*^	–			
12.	T1 Phonological awareness	0.21^**^	0.22^**^	0.31^**^	0.29^**^	0.33^**^	0.36^**^	−0.03	0.05	0.23^**^	0.09	0.19^**^	–		
13.	T1 Rapid naming	0.17^**^	0.06	0.11	0.06	0.27^**^	0.25^**^	−0.07	0.13^*^	0.24^**^	−0.08	0.12^*^	0.17^**^	–	
14.	T1 Nonverbal intelligence	0.21^**^	0.23^**^	0.24^**^	0.27^**^	0.27^**^	0.25^**^	0.05	0.16^**^	0.24^**^	0.21^**^	0.19^**^	0.30^**^	0.06	–

T1 represents time 1; T2 represents time 2.

* *p* < 0.05;

** *p* < 0.01.

**Table 3:** Model fit indexes and model comparisons.

Model	*χ* ^2^	*df*	*p*	Comparative fit index	Tucker Lewis index	Root mean square error of approximation	Standardized root mean square residual	Satorra–Bentler scaled *χ*^2^ difference test		
1	4.208	2	0.12	0.996	0.934	0.06	0.01	∆*χ*^2^	∆*df*	*p*
2	8.942	3	0.03	0.990	0.881	0.08	0.01	4.705	1	0.03
3	7.215	3	0.07	0.993	0.916	0.07	0.01	3.039	1	0.08

Model 1 represents the full model. Model 2–3 represent the alternative models. The path from T1 morphological meaning analysis to T2 word reading was removed in model 2, and that from T1 general morphological knowledge to T2 word reading was removed in model 3. T1 represents time 1; T2 represents time 2.

### Testing and Comparison of Autoregressive Models for Research Question 1

We explored the research questions by using autoregressive path models. Mplus 7.0 (Muthén and Muthén, 2017) was employed to fit our path models. The full-information maximum likelihood was used to account for a small amount of missing data (<0.13% across measures) and to guard against bias from non-normality and non-independence of observations (Finney and DiStefano, 2013). The chi-square statistical test, comparative fit index (CFI), Tucker–Lewis index (TLI), root mean square error of approximation (RMSEA), and standardized root mean residual (SRMR) were used as the indices of model fit. CFI and TLI values at approximately or greater than 0.95 and RMSEA and SRMR estimates close to 0.06 are indicative of good model fit (e.g., Hu and Bentler, 1999). We generated a 95% bias-corrected and accelerated confidence interval (CI) for the indirect effect of general morphological knowledge on word reading through morphological meaning analysis on the basis of 5,000 bootstrapped samples.

**Figure 1:**
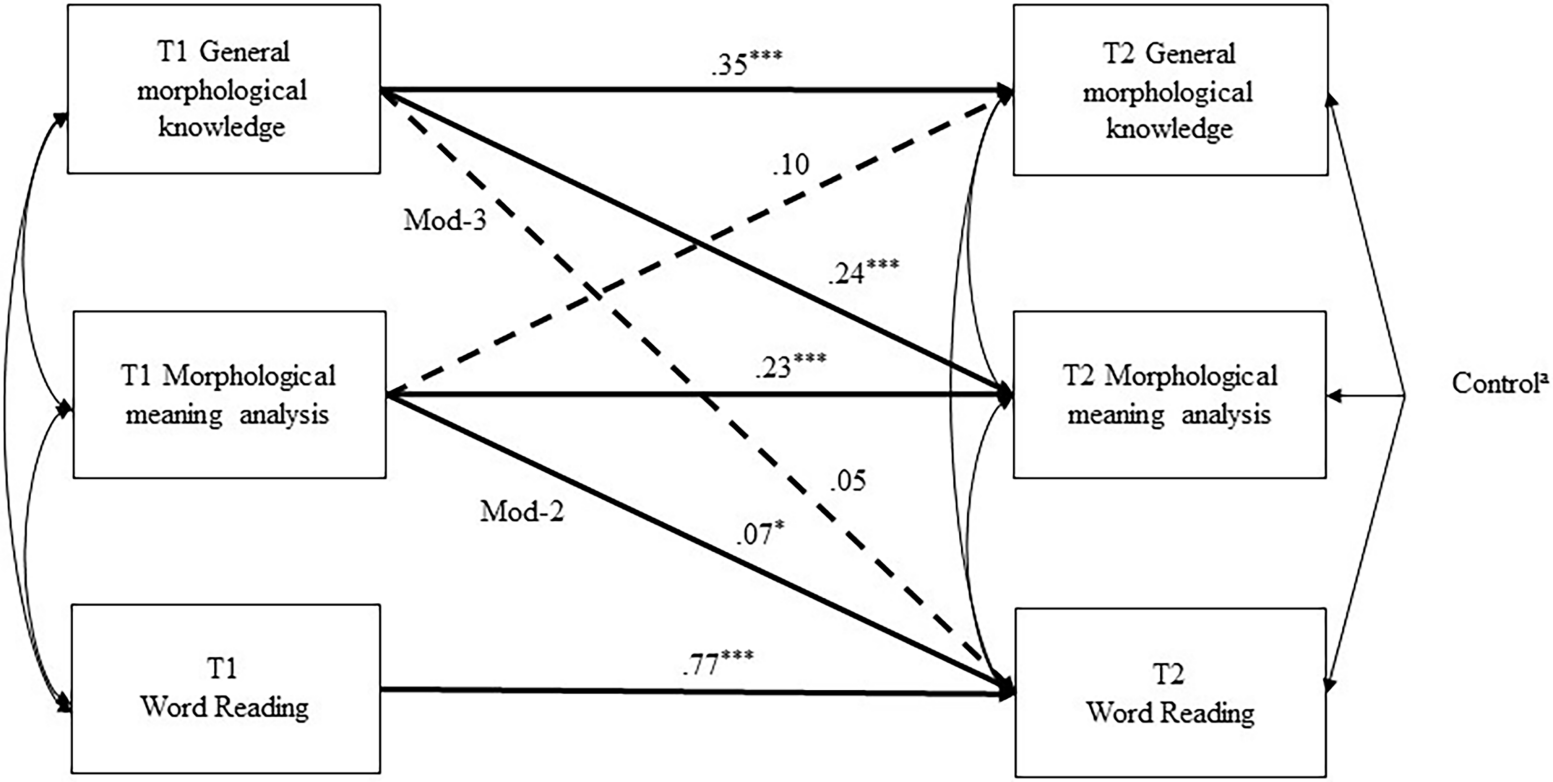
Model 1 with Standardized Coefficients for Paths Note. ^a^All control variables at time 1 included gender and T1 age, vocabulary, orthographic awareness, radical awareness, phonological awareness, rapid automatized naming, nonverbal intelligence. Time 2 general morphological knowledge regressed on gender (*β* = 0.03), age (*β* = −02), vocabulary (*β* = 0.13^*^), orthographic awareness (*β* = 0.03), radical awareness (*β* = 0.09^*^), phonological awareness (*β* = 0.06), rapid automatized naming (*β* = −0.07) and nonverbal intelligence (*β* = 0.09). The predictions of the eight controls on T2 morphological meaning analysis were *β* = −0.02, *β* = −0.04, *β* = 0.14^*^, *β* = −08, *β* = 0.03, *β* = 0.11^*^, *β* = −0.07, *β* = 0.12^*^. The predictions of the eight controls on T2 word reading were *β* = −0.02, *β* = 0.01, *β* = 06, *β* = −0.02, *β* = −0.01, *β* = 06^*^, *β* = −0.01, *β* = −0.01. Mod-2 and Mod-3 paths represent the paths that were individually removed for the testing of nested models. The dotted lines represent the nonsignificant paths. ^*^p < 0.05, ^***^p < 0.001. T1 represents time 1; T2 represents time 2.

To examine the first research question on which MA components could independently contribute to word reading development, we tested the full model (model 1) and the theoretically driven nested models (model 2 and 3). We began with model 1 (see Figure 1) which represented the full model with all the predicted paths included. The model included the autoregressive paths, linking T2 (time 2) MA components and word reading to their respective T1 (time 1) measurements. Considering the close relation between the two MA components, existing paths also showed T1 general morphological knowledge predicting T2 morphological meaning analysis and T1 morphological meaning analysis predicting T2 general morphological knowledge. In addition, T2 outcomes were regressed on control variables of T1 age, vocabulary, orthographic awareness, radical awareness, phonological awareness, RAN, nonverbal ability, and gender. Specifically, the model depicted the possibility that both T1 general morphological knowledge and morphological meaning analysis contribute to T2 word reading.

Across multiple indicators shown in Table 3, model 1 provided a good fit to the data. Figure 1 includes the standardized coefficients for the paths in model 1. We then tested two alternative models (i.e., model 2 and model 3), which showed that only general morphological knowledge or morphological meaning analysis predicted word reading development. We conducted Satorra–Bentler scaled chi-square difference tests (S–B tests) to compare the fit of each nested model to the full model (model 1), the results of which are shown in Table 3. If a significant reduction in model fit was found in the comparison test, then the specific path missing in the nested model represented an important effect.

In model 2, the path from morphological meaning analysis on word reading (Mod-2) was removed from model 1. Therefore, this model tested if only general morphological structure independently predicted word reading development. Comparing the model fit of model 1 and that of model 2 could evaluate the importance of the path between T1 morphological meaning analysis and T2 word reading in model 1. The result of the S–B test showed that the model fit was significantly reduced in model 2 compared with that in model 1 (*p* < 0.05). Thus, it suggested that the path between T1 morphological meaning analysis and T2 word reading played an important role, and should be included in the model.

In model 3, we removed the path (Mod-3) between general morphological knowledge and word reading in model 1 so that the model tested if only T1 morphological meaning analysis predicted T2 word reading. The S–B test revealed no significant difference in the model fit in model 1 and that in model 3 (*p* > 0.05). The finding indicated that removing the path between T1 general morphological knowledge and T2 word reading did not significantly affect the model fit. Thus, general morphological knowledge does not appear to contribute additional independent variance to word reading as shown in Figure 1 Mod-3 path (dotted line).

**Figure 2:**
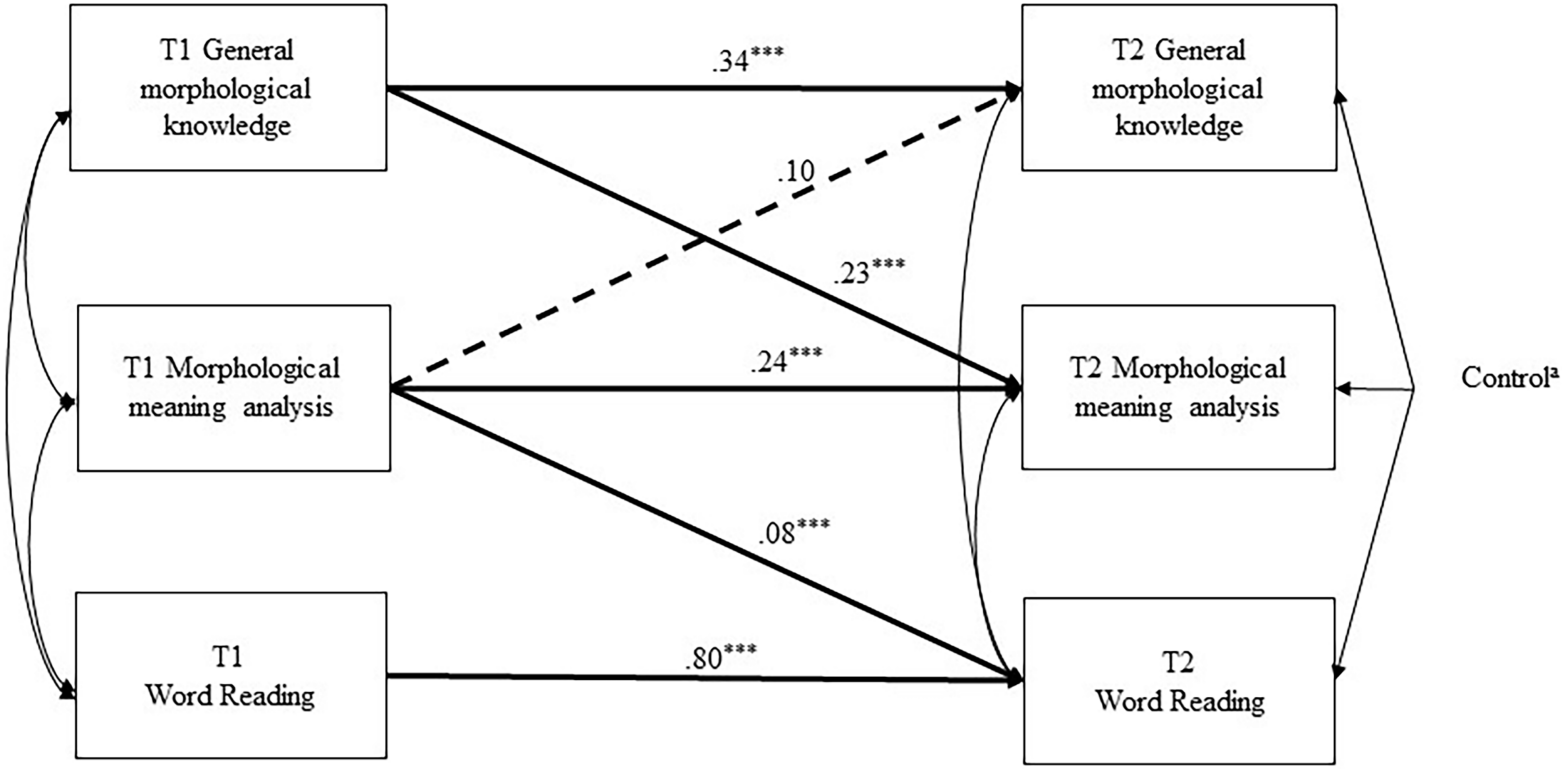
Model 3 with Standardized Coefficients for Paths Note. ^a^All control variables at time 1 included gender and T1 age, vocabulary, orthographic awareness, radical awareness, phonological awareness, rapid automatized naming, nonverbal intelligence. Time 2 general morphological knowledge regressed on gender (*β* = 0.03), age (*β* = −0.02), vocabulary (*β* = 0.14^*^), orthographic awareness (*β* = −06), radical awareness (*β* = 0.09^*^), phonological awareness (*β* = 0.06), rapid automatized naming (*β* = −0.07) and nonverbal intelligence (*β* = 0.09). The predictions of the eight controls on T2 morphological meaning analysis were *β* = −0.02, *β* = −0.04, *β* = 0.14^*^, *β* = −08, *β* = 0.03, *β* = 0.12^*^, *β* = −0.07, *β* = 0.12^*^. The predictions of the eight controls on T2 word reading were *β* = −0.02, *β* = 0.01, *β* = 06, *β* = −0.02, *β* = −0.01, *β* = 06^*^, *β* = −0.01, *β* = −0.01. Mod-2 and Mod-3 paths represent the paths that were individually removed for the testing of nested models. The dotted lines represent the nonsignificant paths. ^*^p < 0.05, ^***^p < 0.001. T1 represents time 1; T2 represents time 2.

A comparison of the fit indexes of the full model and nested models (shown in Table 3) showed that model 3 was the best fitting and more parsimonious model. Figure 2 includes the standardized coefficients for the paths in model 3. All the autoregressive effects of the MA components and word reading were significant, implying that the T1 constructs were highly predictive of themselves at T2. For the cross-lagged paths, T1 general morphological knowledge had a significant moderate effect on T2 morphological meaning analysis, and the prediction from T1 morphological meaning analysis on T2 general morphological knowledge was nonsignificant. In particular, T1 morphological meaning analysis had a significant effect on T2 word reading, which showed that morphological meaning analysis in the two components could independently predict word reading gains after controls. The absence of a path between T1 general morphological knowledge and T2 word reading suggested that general morphological knowledge could not predict word reading gains independently of morphological meaning analysis and other control variables.

### *Post hoc* Mediation Analysis for Research Question 2

In model 3, T1 general morphological knowledge predicted gains in morphological meaning analysis, and T1 morphological meaning analysis predicted gains in word reading over time. This pattern of results supported the hypothesis about the mediating role of morphological meaning analysis in the relation between general morphological knowledge and word reading development. To explore the second question about the mediator of morphological meaning analysis, we performed a *post hoc* mediation test which were assumed to be appropriate for the two-wave longitudinal data (Cole and Maxwell, 2003). The estimate of mediation was generated by multiplying the parameters of the path from T1 general morphological knowledge on T2 morphological meaning analysis, and that from T1 morphological meaning analysis on T2 word reading. Results showed that the indirect effect from T1 general morphological knowledge on T2 word reading *via* morphological was significant (*β* = 0.02; *p* < 0.05; bias-corrected bootstrapped 95% CI [0.004, 0.050]), supporting that morphological meaning analysis played an intermediate role in the relation between general morphological knowledge and word reading.

## Discussion

The study took advantage of a short-term longitudinal design to examine which MA components—general morphological knowledge, morphological meaning analysis, or both—independently predicted word reading half a year later. After partialling out the control variables such as vocabulary, morphological meaning analysis rather than general morphological knowledge could independently predict primary school children’s word reading, whereas general morphological knowledge could only predict word reading indirectly *via* the mediator of morphological meaning analysis. The findings not only confirm the independent contribution of MA to Chinese word reading development, but also support the orthographic–semantic pathway that MA could promote words’ semantic representations and subsequently facilitate word reading performance.

### Impact of Morphological Meaning Analysis on Chinese Word Reading

As results showed, the meaning-analysis morphological retrieval ability significantly predicted word reading independently of the autoregressive effect and control variables. First, this finding confirms the influence of morphological meaning analysis on word reading development independently of vocabulary. Morphological meaning analysis is an online meaning-inference ability, whereas vocabulary represents the stored word semantic knowledge instead of the semantic analysis process (Deacon et al., 2017; Levesque et al., 2017). In the present morphological meaning analysis task, children may not be able to successfully finish the task which required to analyze the semantic relations between words and constitute morphemes, even though they have the stored meanings of the high-frequency word items in their vocabulary. The difference between MA and vocabulary could help explain the result that morphological meaning analysis predicted word reading independently of vocabulary.

Second, consistent with earlier findings that only morphological meaning analysis could enhance reading comprehension (Deacon et al., 2017; Levesque et al., 2019), the result showed that only morphological meaning analysis, instead of general morphological knowledge, could independently predict Chinese word reading development, at least for middle-grade primary-school students. The finding may be due to the fact that, rather than a form of meta-cognitive functioning of general morphological knowledge, morphological meaning analysis directly acts on words’ semantic representations and subsequently improves word reading. For example, children with general morphological knowledge could recognize that the novel word “雪刷 (snow brush)” has the subordinate structure, and the first morpheme modifies the second one. However, they could only rely on morphological meaning analysis to conduct semantic analysis and finally know that the accurate meaning of “雪刷” is “a brush used to remove snow,” not “a brush made of snow.” Morphological meaning analysis, as a significant predictor of word reading development, suggests that MA could foster word reading by working directly on semantic representations.

Previous studies found inconsistent conclusions on whether MA could independently contribute to Chinese word reading beyond the control of vocabulary (e.g., Liu et al., 2013; Hulme et al., 2019). Critically, few of these studies have assessed the influence of morphological meaning analysis on Chinese reading acquisition. Instead, they mainly adopted the measures of general morphological knowledge, which could not help children obtain words’ meanings as accurately as morphological meaning analysis. This reason may explain why the results were unstable that MA could independently predict Chinese word reading. The present study adopted high-frequency words containing homographs as task items to measure morphological meaning analysis, and found that this MA component played a significant role in children’s Chinese reading development. The results supported the feasibility of conducting an accurate assessment of Chinese morphological meaning analysis in primary school students and helped determine which MA components independently contributed to Chinese word reading development.

### Mediating Role of Morphological Meaning Analysis in the Relation Between General Morphological Knowledge and Word Reading

The second goal of the present study was to examine the role of morphological meaning analysis in the relation between general morphological knowledge and word reading. Prior studies found the mediating role of morphological meaning analysis in the correlations between general morphological knowledge and other reading skills (i.e., vocabulary and comprehension; e.g., Zhang, 2015; Levesque et al., 2017, 2019). Similarly, the present results showed that general morphological knowledge could only predict word reading indirectly, and morphological meaning analysis acted as a full mediator between them.

In the relation between the two MA components, the present results supported an assumption from previous studies that general morphological knowledge could predict morphological meaning analysis (Zhang et al., 2016; Levesque et al., 2019). This finding is theoretically reasonable because the increased general morphological awareness, serving as a prior condition of morphological processing, could promote morphological meaning analysis by familiarizing children with utilizing morphological structures in understanding the semantic relations between words and constituent morphemes. Morphological meaning analysis, furthermore, makes children more successful in synthesizing the meanings of multi-morpheme words from single morphemes and thus may improve word reading performance. The mediating effect of morphological meaning analysis on the relation between general morphological knowledge and word reading thus supported again that MA could promote word reading *via* inferring semantic representations.

### Theoretical and Practical Implications of the Present Findings

By dividing MA into two components, the present study showed that MA could contribute to word reading development independently of vocabulary. Specifically, morphological meaning analysis could independently predict word reading, and general morphological knowledge could boost morphological meaning analysis, which, in turn, facilitated word reading development. These findings pointed to a significant role of morphological meaning analysis in word reading development: morphological meaning analysis predicted word reading independently of vocabulary, and it could also connect general morphological knowledge with word reading. The importance of morphological meaning analysis thus supports the orthographic–semantic pathway account that MA contributes to word reading development by facilitating semantic representations (Li and McBride-Chang, 2014; Tong et al., 2017). Future studies could focus on the role of morphological meaning analysis, which is distinct from other word knowledge measures, such as vocabulary, in Chinese word reading.

From a pedagogical perspective, the independent prediction of morphological meaning analysis on word reading development suggests that improving morphological meaning analysis skills would be effective to support children’s Chinese word reading development. For example, morphological interventions about interpreting multi-morpheme-word meanings with knowledge of single morphemes and morphological structures (e.g., Wu et al., 2009) could be applied to promote word reading. Moreover, the mediating effect of morphological meaning analysis implies that increasing children’s general sensitivity of morphological structures (e.g., Zhou et al., 2012) could also enhance word reading by promoting meaning-analysis skills.

### Limitations and Future Directions

The present study has limitations. First, the two waves of data collection with a six-month interval only allowed us to explore short-term word reading gains but not the growth of reading skills. Second, the reliability of the morphological retrieval task, ranging from 0.6 to 0.7 in the present study, was relatively low. The possible reason for the low reliability was that the forced-choice task was prone to guessing effect. Further studies should try to improve the reliability of tasks, for example, by correcting the data for guessing, or by asking children to interpret how they retrieve single-morpheme meanings from whole words. In addition, though morphological meaning analysis played a significant role in Chinese word reading, its role may be somewhat different from that in alphabetic languages. Future research could conduct the cross-language comparative exploration about the roles of MA in word reading skills.

## Conclusion

The current study is the first one to examine the contribution of MA to word reading development by including both general morphological knowledge and morphological meaning analysis in Chinese primary school children. Findings showed that morphological meaning analysis, rather than general morphological knowledge, predicted word reading development independently of vocabulary. Moreover, morphological meaning analysis mediated the relation between general morphological knowledge and word reading development. These findings provide an empirical support for the effect of MA on word reading development independently of vocabulary, and support the orthographic–semantic pathway that MA facilitates Chinese word reading by strengthening words’ semantic representations.

## Data Availability Statement

The raw data supporting the conclusions of this article will be made available by the authors, without undue reservation.

## Ethics Statement

The studies involving human participants were reviewed and approved by University Committee on Human Research Protection. Written informed consent to participate in this study was provided by the participants’ legal guardian/next of kin.

## Author Contributions

HL, JW, LW, and CD contributed to conception and design of the study. HL and JW administered the data collection. HL and WZ performed the statistical analysis. HL wrote the first draft of the manuscript. JW, LW, and CD made important contributions to the revision of the manuscript. All authors contributed to the manuscript and read, edited and approved the submitted version.

## Funding

This study was supported by the Programs Foundation of Shanghai Municipal Commission of Health and Family Planning (201540114), Key Specialist Projects of Shanghai Municipal Commission of Health and Family Planning (ZK2015B01), and Research fund of Jiangsu Provincial Key Constructive Laboratory for Big Data of Psychology and Cognitive Science (72592162006G).

## Conflict of Interest

The authors declare that the research was conducted in the absence of any commercial or financial relationships that could be construed as a potential conflict of interest.

## Publisher’s Note

All claims expressed in this article are solely those of the authors and do not necessarily represent those of their affiliated organizations, or those of the publisher, the editors and the reviewers. Any product that may be evaluated in this article, or claim that may be made by its manufacturer, is not guaranteed or endorsed by the publisher.
